# Enhanced Optical Modulation Properties via Two-step Annealing of Sol–Gel Deposited Vanadium Dioxide Thin Films

**DOI:** 10.1021/acsomega.4c08910

**Published:** 2025-01-20

**Authors:** Zhencheng Li, Jiacheng Yu, Yongde Xia, Zhuxian Yang, Yunbin He, Nannan Wang, Yanqiu Zhu

**Affiliations:** 1 Department of Engineering, Faculty of Environment, Science and Economy, University of Exeter, Exeter EX4 4QF, United Kingdom; 2 School of Materials Science and Engineering, Hubei University, Wuhan 430062, China; 3 School of Resources, Environment and Materials, Guangxi University, Nanning 530004, China

## Abstract

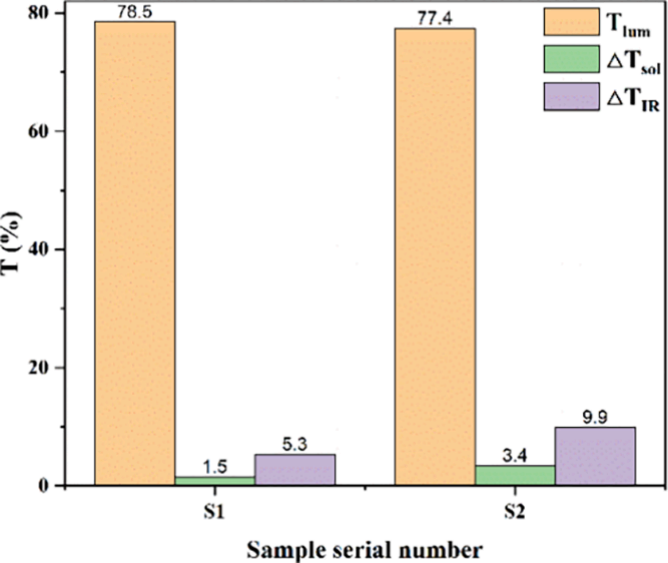

Vanadium dioxide (VO_2_) is a key coating component for smart devices, especially in smart windows, due to its excellent thermochromic properties and metal-insulating transformation. In this study, we investigate the influence of different annealing on the VO_2_ thin films prepared by a sol–gel technique combined with spin-coating and compare the changes in morphology, structure, and optical properties. Of the two types of VO_2_ (M) films obtained by one-step annealing at 700 °C and two-step annealing at 500 and 700 °C, the two-step annealed samples formed an intermediate phase VO_2_ (B), which allowed them to exhibit higher purity and better crystallinity than those of the one-step annealed samples. An in-situ XRD study confirmed that the higher purity and better crystallinity enabled the resulting VO_2_ thin films to lower the phase transition temperature, where the phase transition occurred by 5 °C earlier upon heating and about 10 °C upon cooling. As a result, the infrared and solar modulation capabilities were improved by 87 and 127%, respectively, while the high visible transmittance of the samples essentially remained unchanged. This study has demonstrated that proper annealing can refine the structures of VO_2_ thin films and enhance their optical properties.

## 1 Introduction

Vanadium dioxide (VO_2_) is well-known for its unique metal–insulator phase transition behavior that generates a reversible and significant electrical and optical property change when crossing a critical temperature (*T*_c_) of about 68 °C.^1−3^ This reversible change makes VO_2_ very attractive for applications in sensors,^4^ batteries,^5^ and electronic switches.^6^ In particular, due to its merit in energy saving and gas emission reduction, VO_2_ has received widespread attention for its great potential to be used in smart windows.^7^ Below the *T*_c_, VO_2_ is in a monoclinic phase, VO_2_ (M), which behaves as a semiconductor and allows most infrared light to be transmitted, while above the *T*_c_, VO_2_ turns into a tetragonal phase, VO_2_ (R), which behaves as metallic and blocks the transmission of infrared light. These reversible phase transitions allow VO_2_ (R) to convert back to VO_2_ (M) upon cooling below the *T*_c_.^8,9^ This feature enables VO_2_-based smart windows to have temperature control capabilities and thus to intelligently adjust the room temperature according to the environment, realizing energy saving.

For smart window applications, high-quality VO_2_ films are needed. The quality of the VO_2_ films is directly related to their preparation methods. Many methods have been reported to date due to their respective advantages, such as chemical vapor deposition,^10^ atomic layer deposition,^11^ pulsed laser deposition,^12^ magnetron sputtering,^13^ and the sol–gel method.^14^ For example, the most widely used magnetron sputtering technique could produce VO_2_ films with extremely high uniformity and strong substrate adhesion, but the high costs of expensive equipment and target materials limited its application.^15^ Compared with above dry deposition techniques, wet chemical techniques such as the sol–gel method have the advantages of lower costs, larger deposition areas, and easier subsequent doping, which allows for the production of complex VO_2_ films with more functionalities.^16^

Li et al. prepared VO_2_ films by a stable and low-cost sol–gel method, and by introducing silicon dioxide as an antireflective coating, they have improved the thermochromic properties of the films.^17^ The results showed that the resistivity change rate and transmittance of the VO_2_ films during the metal-insulation phase transition were significantly improved. Panburana et al. prepared solutions using vanadium(V) triisopropoxy oxide (VO(OC_3_H_7_)_3_) as a precursor and investigated the effect of different annealing conditions on the properties of the VO_2_ films.^18^ Their results showed that the film became better with the increase in annealing temperature and annealing time in the range 300–500 °C and 30–120 min. In addition, different substrate materials also affected the film quality, and sapphire (Al_2_O_3_) turned out to be the best substrate for film formation. Guo et al. investigated the structure and phase transition properties of VO_2_ thin films deposited on c-sapphire substrates by the sol–gel method in relation to the annealing time.^19^ Their study showed that prolonging the annealing time helped to improve the quality and related properties of VO_2_ thin films. When the annealing time was 4–7 h, the VO_2_ films would exhibit good phase transition properties (up to three orders of magnitude change in electrical resistance), and the sintered films after 7 h had larger grain sizes, better growth orientation, and lower phase transition temperatures compared to the 4 h sintered films. These studies provided useful information and support for the subsequent VO_2_ doping and kinetic studies.^20,21^ However, due to the close association of VO_2_ polycrystals, VO_2_ films prepared by the sol–gel method generally suffered from low purity and multiphase coexistence, making it difficult to obtain high purity films of highly orientated single crystal phase.^22^ The postgrowth annealing can help promote the quality of VO_2_ films, for which the annealing parameters (time, temperature, pressure, etc.) are particularly important, as they all affect the valence state of vanadium and thus determine the final thermochromic properties. Problems such as sample impurity and poor crystallinity are also present in VO_2_ films prepared by other methods due to the difficulty in obtaining optimal unification of the many annealing parameters.

In this study, we report the preparation of high-purity and highly oriented VO_2_ thin films by a facile and low-cost sol–gel technique that uses vanadium pentoxide powder (V_2_O_5_) as a precursor and deionized water as the solvent. We characterized the morphologies, structures, and optical properties of the resulting films subjected to two-step annealing. We have found that the two-step annealing of the highly pure and crystalline layered VO_2_ (B) as a transition phase eliminated the problems of multiphase coexistence and low crystallinity of most VO_2_ films obtained by the sol–gel method and enhanced the thermochromic properties of the VO_2_ (M) film.

## 2 Experimental Section

### 2.1 Synthesis of VO_2_ Thin Films

VO_2_ thin film samples were prepared from vanadium pentoxide (V_2_O_5_) powder using a sol–gel technique. Two grams of V_2_O_5_ powder (99.6%, Sigma-Aldrich) was placed in an alumina boat and subsequently placed in a furnace for 15 min at 850 °C to melt. The molten V_2_O_5_ was quickly poured into 80 mL of deionized water in a beaker at 60 °C to obtain the sol and then stirred vigorously at 60 °C for 5 h and left overnight to obtain a dark brown V_2_O_5_ gel, which was used to prepare the initial V_2_O_5_ film.

The V_2_O_5_ film samples were prepared on fused quartz slides (25 × 25 mm^2^, 1 mm thickness, UQG Optics) by spin-coating. The quartz slides were precleaned with acetone, alcohol, and deionized water and then dried before use. The entire coating process was divided into two steps; the first step was to rotate at 400 rpm for 10 s, followed by the second step with the spinning speed increased to 3000 rpm for 20 s. The V_2_O_5_ gel was diluted with deionized water in a 1:1 ratio. Three to five drops of the diluted solution were applied to the quartz substrate adhered to the spin-coater in the first step of coating, and the V_2_O_5_ film was obtained after the entire spin-coating process. The resulting initial V_2_O_5_ films were then transferred to a hot plate for drying at 75 °C for 1 h. Different thicknesses of the films could be controlled by adjusting the dilution ratio of the solution and the actual number of drops applied, at a fixed routine of spinning speed.

By annealing the above V_2_O_5_ films, we obtained VO_2_ films of different types. The first type was obtained directly by a one-step annealing treatment, as a VO_2_ (M) film, named as Sample 1 (S1). The annealing was carried out in a tube furnace under an Ar atmosphere at 700 °C for 2 h and then naturally cooled to room temperature to obtain S1. The second type was obtained via a two-step annealing, as a VO_2_ (M) film, named Sample 2 (S2). The first step annealing was carried out in a tube furnace under an Ar atmosphere at 500 °C for 1.5 h and naturally cooled to room temperature to obtain a VO_2_ (B) film, which is an intermediate phase in the process of the preparation of a VO_2_ (M) film. The resulting VO_2_ (B) film was followed by the second step annealing at 700 °C under Ar for another 2 h and naturally cooled to room temperature, resulting in S2. The heating rates of the tube furnace for all processes were 10 °C/min.

### 2.2 Characterization of VO_2_ Thin Films

The thickness and surface morphology of all VO_2_ films were characterized using a focused ion beam scanning electron microscope (FIB-SEM, xT Nova Nanolab 600 FIB), operated at 10 kV for the SEM mode and 30 kV for the FIB mode. The high-resolution images and lattices of the VO_2_ films were recorded by a transmission electron microscope (TEM, JEOL 2100 TEM), operated at 200 kV. For the TEM characterization, different VO_2_ films were carefully scraped off the substrates and then dispersed in acetone under ultrasonication for 20 min. The suspension was dropped onto a carbon-coated Cu grid to create the TEM specimen. The phase transition and structure of the VO_2_ films were studied by an X-ray diffractometer (XRD, Bruker D8 advanced XRD) equipped with an in-situ high-temperature thin film test kit, using a Cu Kα radiation λ = 0.15418 nm and a scanning step size of 0.03°, operated at 40 mA and 40 kV. The in-situ high temperature measurement started at 25 °C and ended at 95 °C, with an interval temperature of 10 °C and a heating rate of 5 °C/min, and at each data collection point, the temperature was held for 40 min. The elemental and valence states of the VO_2_ films were characterized using X-ray photoelectron spectroscopy (XPS, Axis Ultra DLD), with an Al Kα radiation and energy scan range from 0 to 1380 eV. The transmittances and optical properties of the VO_2_ films were investigated on a UV–vis–NIR spectrophotometer (UV-3600 Plus, Shimadzu), with a wavelength from 250 to 2500 nm, at 25 and 90 °C, respectively.

## 3 Results and Discussion

**Figure 1:**
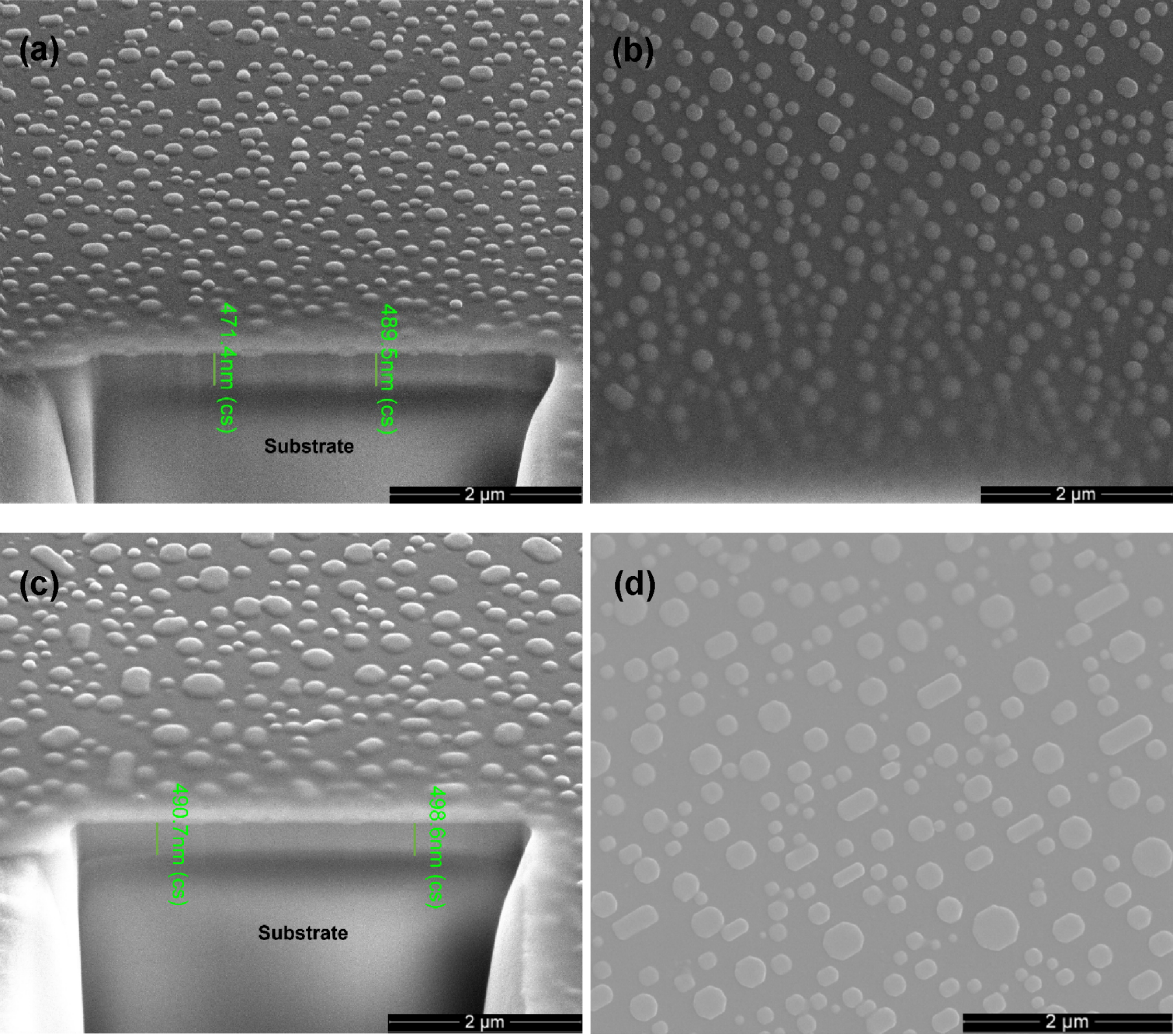
SEM images of VO_2_ films obtained from different annealing processes. (a) Cross-sectional and (b) top views of S1. (c) Cross-sectional and (d) top views of S2.

Figure 1 shows the SEM images of the cross-sectional and top views of different VO_2_ film samples. The cross-sectional views were taken at a tilt angle of 52° from the top surface of the sample where the small holes were dug out by the FIB. The average film thicknesses of S1 and S2 are measured 480 and 495 nm, respectively (Figures 1a and 1c). Both films are of good quality, with a dense and uniform internal structure without cracks or gaps, tightly adhered to the substrate. The top views in Figure 1b,d show regularly shaped particles on the surfaces of both samples. The sizes of these particles vary from tens to hundreds of nanometers and are randomly distributed on the surface of the films in the form of isolated islands. It is very likely that these islands were formed due to the ripening and agglomeration of the surrounding pristine fine VO_2_ grains at high temperatures during the heat treatment and annealing process. Moreover, the average grain size on the surface of S2 obtained from the two-step annealing (Figure 1d) is significantly larger than that of S1 (Figure 1b), appearing as regular squares, octagons, or prisms against the small spherical particles in S1.

**Figure 2:**
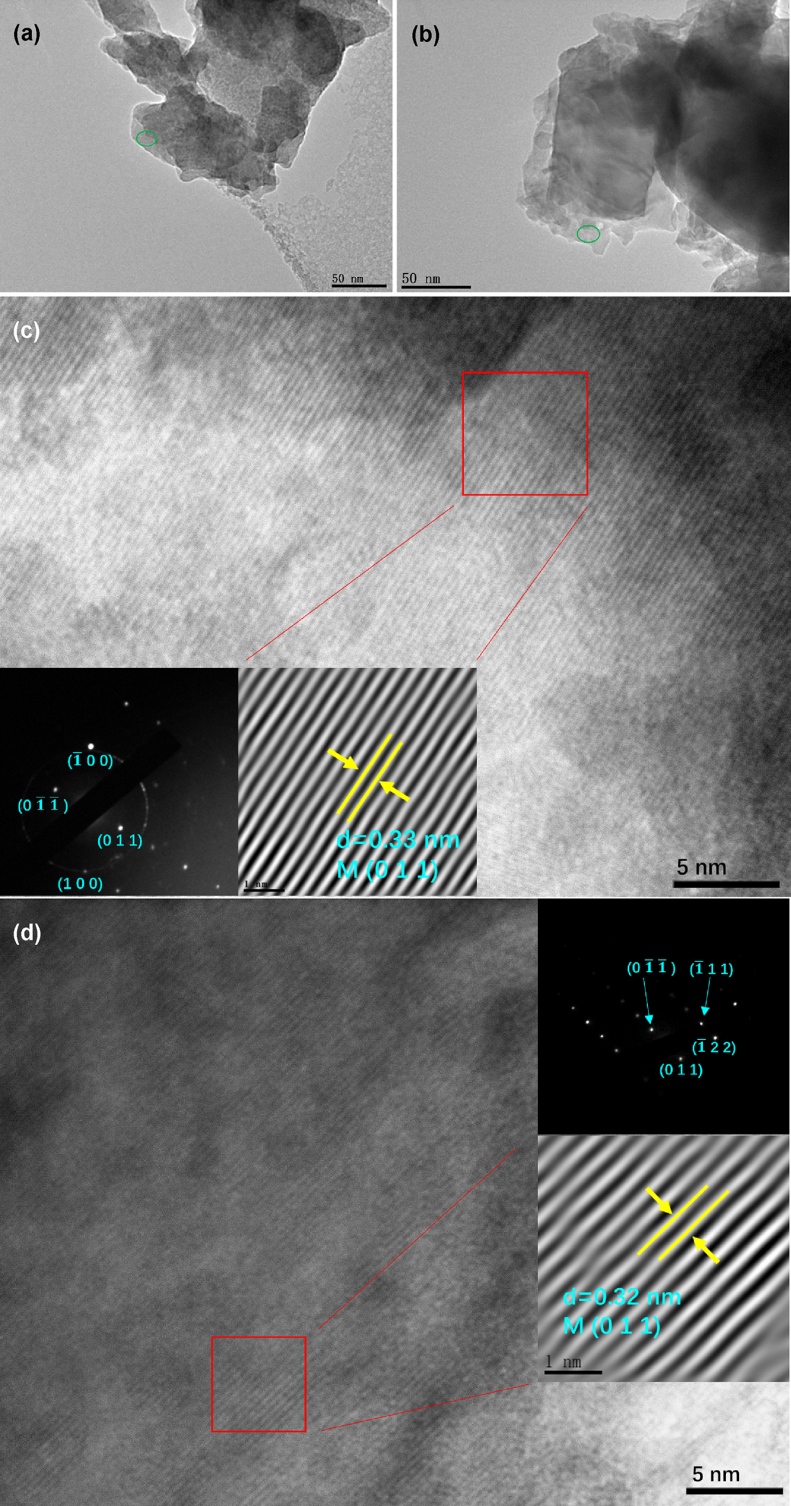
TEM images of VO_2_ films prepared by different annealing processes. (a, b) Low-magnification images of S1 and S2. (c, d) High-resolution images of the green circled regions of S1 and S2. The insets show their corresponding selected area electron diffraction (SAED) patterns and inverse Fourier transform (FFT) diffractograms of the red square areas.

Figure 2 shows the bright-field TEM images of the heat-treated VO_2_ films. At lower magnifications (Figure 2a,b), the samples consist of several stacks of flattened fragments of significantly varied thicknesses, with sizes ranging from tens to hundreds of nanometers. At higher resolution (Figure 2c,d), both samples clearly exhibit sharp lattice fringes, matching well with monoclinic crystalline structures of VO_2_ (M), which are further confirmed by the insets showing the SAED patterns and inverse FFT diffractograms. The interplanar spacings of S1 and S2 are 0.33 and 0.32 nm, respectively, corresponding to the (011) plane of the monoclinic VO_2_ (M). It is worth noting that the interplanar lattice spacing of (011) in S2 is slightly smaller compared with S1, which may be attributed to further shrinkage of the lattice due to the internal stress release induced by the secondary annealing. This results in a more regular lattice arrangement of the partially deformed or distorted lattice after the first annealing, with smaller lattice values and improved quality of atomic arrangement.

**Figure 3:**
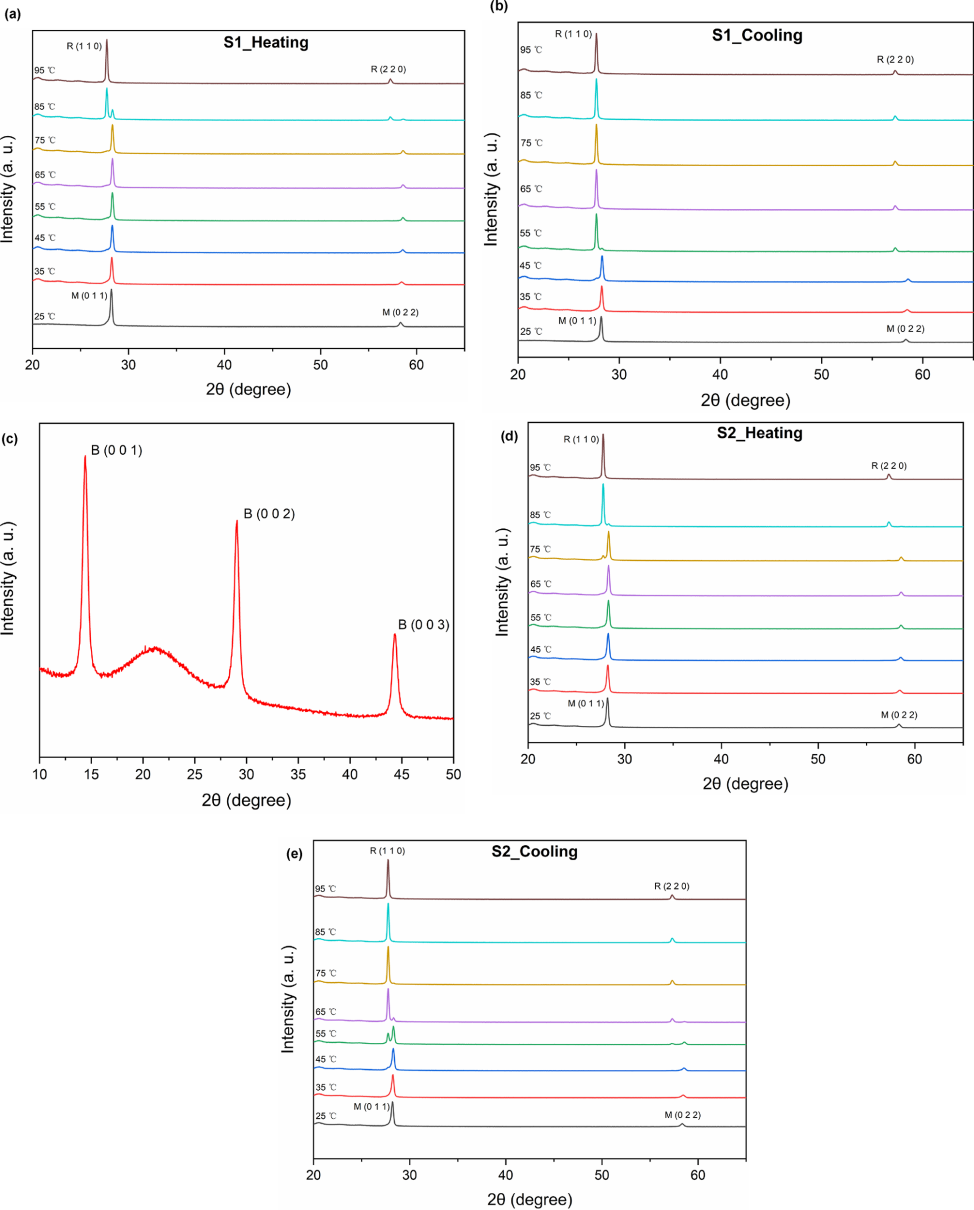
XRD patterns of the VO_2_ films obtained from different annealing processes. In-situ XRD profiles of S1 (obtained from the one-step annealing) for the heating process (a) and the cooling process (b). (c) XRD pattern of the intermediate phase VO_2_ (B) obtained after the first-step annealing for S2 and the in-situ XRD profiles of sample S2 (obtained from the two-step annealing) for the heating process (d) and the cooling process (e).

Figure 3 shows the in-situ XRD profiles of different VO_2_ film samples. At room temperature (25 °C), two sharp peaks were observed at 2θ of 28.2 and 58.4° for both S1 and S2, corresponding to the (011) and (022) planes of the monoclinic VO_2_ (M), respectively, which matched with JCPDS Card No. 43-1051. At a high temperature (95 °C), the two sharp peaks appearing at 2θ of 27.7 and 57.3° correspond to the (110) and (220) planes of the tetragonal rutile VO_2_ (R), respectively (JCPDS Card No. 76-0675). Here, the VO_2_ (M) peaks produced an overall slight shift to a higher angle, which could be attributed to the combined effects of stress and temperature in the films.^23^ Since the particle size of the films obtained by annealing in Ar was much smaller than that obtained by annealing in vacuum, this could lead to compressive stresses in the in-plane direction, which would result in lattice shrinkage and narrowing of the interplanar spacing. The irregular broad peaks located around 2θ of 21° in Figure 3a,b,d,e were raised due to the temperature-controlled test kit for thin films that came along with the XRD equipment and the substrate. Figure 3c shows the XRD pattern of the intermediate phase VO_2_ (B) obtained after the first-step annealing, with three sharp peaks at 2θ of 14.4, 29.1, and 44.3° matching with the (001), (002), and (003) planes of the monoclinic VO_2_ (B) (JCPDS Card No. 81-2392). The broad hump at around 2θ of 21° originated again from the substrate. The VO_2_ (B) was converted to VO_2_ (R) by further heat treatment, and when the VO_2_ (R) naturally cooled to room temperature, it yielded VO_2_ (M) for S2.^24^ It can be found that both S1 and S2 exhibited highly preferential orientations, with (00*l*), (0k*l*), and (*hk*0) being the dominant planes for VO_2_ (B), VO_2_ (M), and VO_2_ (R), respectively. This strong preferential orientation was related to the film thickness and was generally a result of the combination of the internal film stresses and surface energies.^25^ The absence of other peaks in the XRD profiles revealed the high quality and purity of the samples. It is worth pointing out that the peaks of S2 were shifted to the right by a very small angle (∼0.03°) compared with those of S1, which confirmed that the interplanar spacings of S2 were slightly reduced compared to those of S1, verifying its better crystallinity.

During the heating process, the (011) peak of VO_2_ (M) in S1, as shown in Figure 3a, remained stable until the temperature was raised to 85 °C when the (110) peak representing VO_2_ (R) appeared and gradually strengthened. At the same time, the intensity of the (011) peak of VO_2_ (M) weakened and then completely disappeared at around 95 °C, which indicated the completion of the phase transition from VO_2_ (M) to VO_2_ (R). However, during the cooling process, as shown in Figure 3b, the critical temperature of the phase transition dropped significantly, and this difference was most likely due to supercooling. The (011) peak representing VO_2_ (M) did not appear until the temperature dropped to 55 °C and gradually strengthened, accompanied by the gradual weakening of the (110) peak of VO_2_ (R) until it disappeared at 45 °C, which indicated the completion of the reversion from VO_2_ (R) to VO_2_ (M). These results revealed that for S1, the phase transition of the VO_2_ (M) film during heating started from 75 °C and ended at 95 °C, with dominant transitions occurring between 80 and 90 °C. For the cooling process, the phase transition occurred at 45–55 °C. Similarly, for S2 (Figure 3d,e), the (110) peak of VO_2_ (R) began to appear at around 75 °C for heating, whereas the original (011) peak of VO_2_ (M) almost disappeared at about 85 °C, which indicated a phase transition temperature range of 75–85 °C for the heating process. For the cooling process, the (011) peak of VO_2_ (M) started to appear when it was cooled to 65 °C, and the original (110) peak of VO_2_ (R) disappeared at about 45 °C, indicating a wider phase transition range of 45–65 °C.

Compared with S1, S2 has a lower critical temperature for the phase transition during heating, with a difference of about 5 °C. For the reversed phase transition during cooling, the phase transition also started earlier, and its temperature range expanded from 10 to 20 °C. These changes are most likely due to the microstructure differences caused by the different annealing processes, as the secondary annealing resulted in a further improvement in the crystallinity and grain orientation of the VO_2_ films. Since the unoriented grains in the films act as a barrier to the phase transition process, the fewer they are, the less additional thermal energy is required to overcome the energy barrier and the faster the temperature responses during the phase transition.^26^

**Figure 4:**
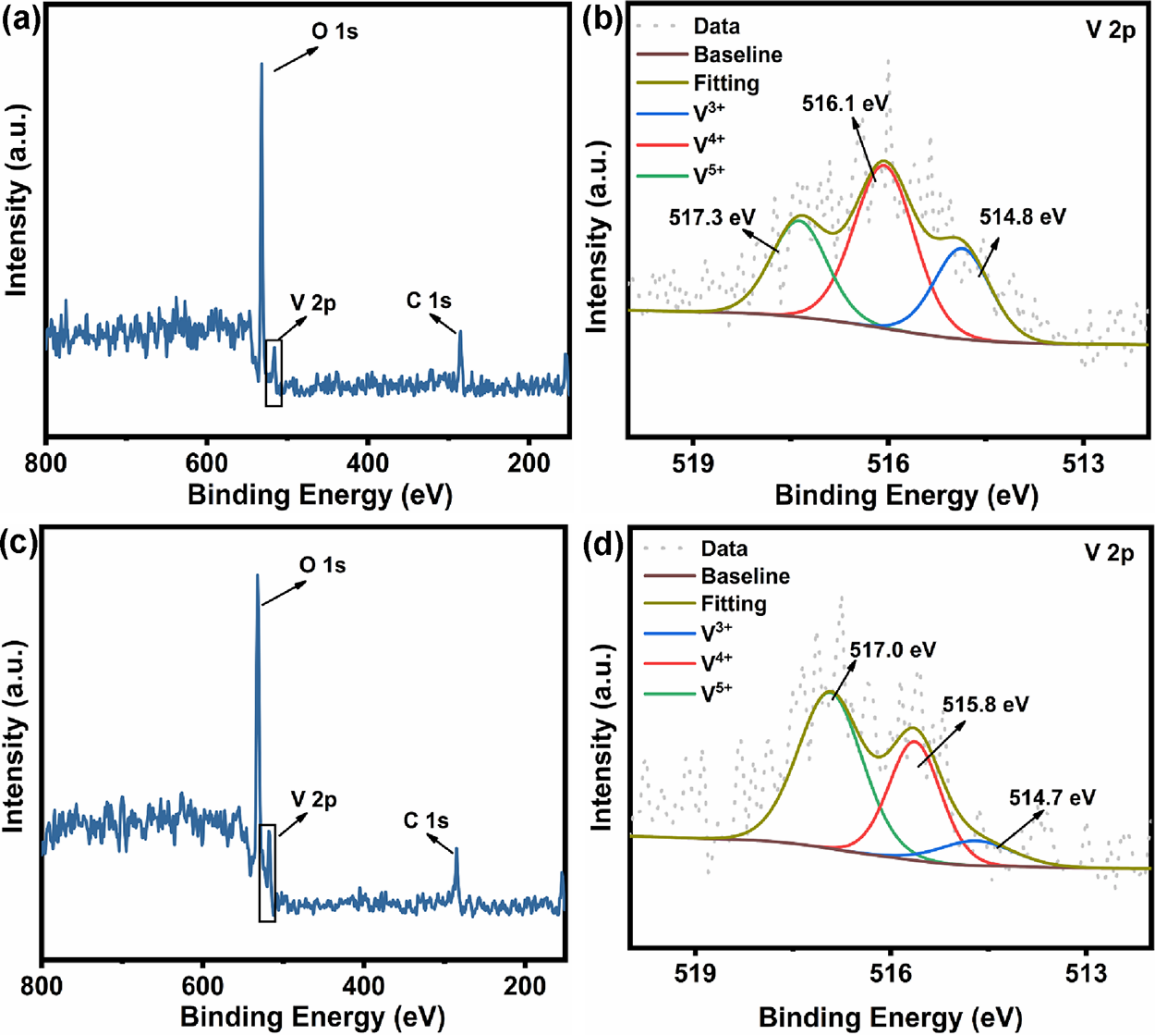
XPS spectra of the VO_2_ films prepared by different annealing processes. (a) XPS survey scans and (b) high-resolution V 2p_3/2_ core energy level deconvolution spectrum of S1. (c) XPS survey scans and (d) high-resolution V 2p_3/2_ core energy level deconvolution spectrum of S2.

XPS was used to characterize the main chemical composition and the valence states of the two VO_2_ thin film samples, the results of which are shown in Figure 4. Known from the survey scans in Figure 4a,c, both S1 and S2 contain three elements: C, V, and O. The adventitious C 1s peak with an energy of 284.8 eV was used to calibrate the binding energy of V. Figures 4b and 4d show the high-resolution scans and deconvolution analyses of the V 2p_3/2_ core energy level spectra of S1 and S2, respectively. The peak of V 2p_3/2_ can be deconvoluted into three peaks corresponding to the V^5+^, V^4+^, and V^3+^ valence states where vanadium is present in different proportions. This is reflected in S1 as three peaks located at 517.3 516.1, and 514.8 eV, respectively (Figure 4b). Similarly, in S2 these three peaks are located at 517.0 515.8, and 514.7 eV, respectively (Figure 4d). All three valence states were present in both VO_2_ thin film samples, with V^4+^ being the highest content in S1 and changing to V^5+^ in S2. This change can be attributed to the overoxidation of the VO_2_ samples on the film surface resulting from the two-step annealing process.^27^ The presence of V^5+^ implies the presence of V_2_O_5_ on the surface of the samples, which is the most preferred VO_*x*_ compound to be formed under atmospheric conditions.^28^ Since the XPS measurement technique is mainly focused on the surface composition of the film, which is characterized at a thickness of no more than 10 nm,^29^ the V_2_O_5_ attached to the surface due to the multistep heating is easily detected, while the film interior is still probably dominated by VO_2_. Furthermore, as oxygen atoms are adsorbed on the surface and attempt to diffuse into the interior of the film during the oxidation process, the internal vanadium atoms are also attempting to diffuse upon the surface of the film; thus, the V^5+^ ions on the surface of the film are constantly being consumed, which leads to the mixing of vanadium ions of different valence states on the surface of the film, and it is difficult to form a single valence state.^30,31^ In addition, we also observed that the content of V^3+^ in S2 was drastically reduced to almost disappeared. This indicated that the purity of the VO_2_ sample was further improved due to the two-step annealing process, and V_2_O_3_ was almost completely converted into VO_2_ and V_2_O_5_.

**Figure 5:**
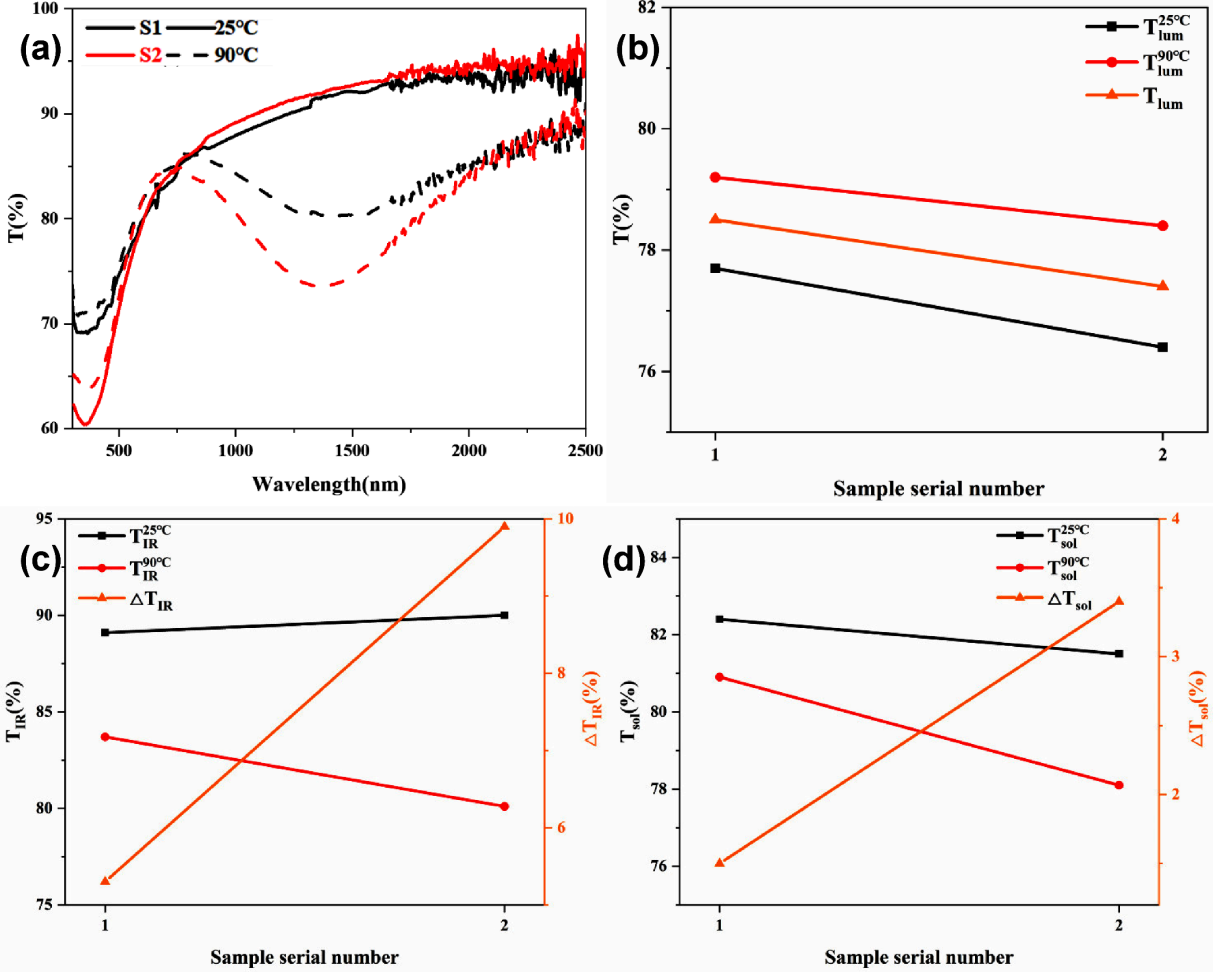
(a) UV–vis–IR optical transmittance spectra of VO_2_ films prepared by different annealing processes at 25 and 90 °C. (b) Integral luminous transmittance (*T*_lum_: covering wavelength of 380–780 nm) at 25 and 90 °C. (c) Integral infrared light transmittance (*T*_IR_: covering wavelength of 780–2500 nm) and (d) solar transmittance (*T*_sol_: covering wavelength of 300–2500 nm) at 25 and 90 °C and the corresponding modulation capacity. The sample serial numbers 1 and 2 shown in (b), (c), and (d) correspond to S1 and S2, respectively.

Figure 5a shows the transmittance of the UV–vis–IR spectra of the two VO_2_ film samples at room temperature (25 °C) and a high temperature (90 °C). It can be found that both S1 and S2 experienced significant transmittance changes at high temperatures, which was caused by the metal-insulating phase transition of VO_2_. At room temperature, the transmittance in the visible wavelength range (taking λ = 550 nm as an example) of both S1 and S2 remained above 70%, indicating a good visible transparency, which rose with the increase in wavelength up to more than 90% in the whole visible-infrared wavelength range. At high temperatures, this trend was significantly different in the infrared wavelength range 780–2500 nm. The transmittance gradually decreased with the increase in wavelength, with the lowest decreasing to about 80 and 72% for S1 and S2, respectively, which was due to the function of VO_2_ (R). It is noteworthy that compared with S1, S2 showed a lower transmittance at a high temperature by about 8% in the infrared light region. The reduction in infrared light transmittance of 8% indicates that the infrared modulation capability of S2 has been greatly improved while keeping the visible transmittance basically unchanged.

It is well-known that solar irradiance varies with wavelength. Therefore, we quantitatively evaluated the thermochromic performance of the samples based on simulated air mass (AM) 1.5 solar irradiance spectra. According to the recorded transmittance spectrum, the following equations (eqs 1–4) were used to calculate the luminous transmittance (*T*_lum_), infrared transmittance (*T*_IR_), and solar transmittance (*T*_sol_):^32^
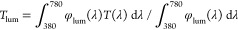
1
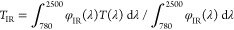
2
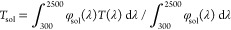
3
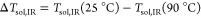
4where φ_lum_(λ) is the spectral sensitivity of the human naked eye, φ_IR_(λ) is the spectral sensitivity of integral infrared light, and φ_sol_(λ) is the spectrum of solar irradiance (AM 1.5). *T*(λ) is the recorded film transmittance at wavelength λ. The integrated wavelength ranges of *T*_lum_ are 380–780 nm and 780–2500 nm for *T*_IR_ and Δ*T*_IR_ and 380–2500 nm for *T*_sol_ and Δ*T*_sol_. In eq 4, 25 and 90 °C represent the test temperatures for all samples.

Figure 5b shows the *T*_lum_ values of S1 and S2 at 25 °C (*T*_lum_^25°C^) and 90 °C (*T*_lum_^90°C^), respectively, as well as the integral transmittances of the two films. Compared with S1, the transmittance of S2 was slightly reduced (from 78.5 to 77.4%); however, it still had a very high transmittance of visible light. As shown in Figure 5c,d, S2 shows significant enhancement in both Δ*T*_IR_ and Δ*T*_sol_ compared to S1, with performance improvements of 87% (from 5.3 to 9.9%) and 127% (from 1.5 to 3.4%), respectively. This indicates that S2 can optimize Δ*T*_IR_ and Δ*T*_sol_ while keeping *T*_lum_ essentially unchanged.

It was observed that VO_2_ films still have high transmittance in the UV region at wavelengths around 300 nm, and this phenomenon may be related to the structure and energy state of the film.^33^ For the thin film structure, multiple reflections and interferences of light can lead to enhanced light transmission at certain wavelengths, and some defect states may be located in the bandgap, affecting the electron jumping behavior and making the material less capable of absorbing light at specific wavelengths.^34,35^ Despite the small bandwidth of VO_2_ (∼0.7 eV), electron-excitation processes in the high-energy UV region (below 300 nm wavelength) are likely to involve electronic states of higher energy levels, which can significantly reduce the absorptive capacity of the film if the limited density of these high-energy excited states prevents them from efficiently absorbing these UV photons.^36^ All of these factors may weaken the UV absorption effect of VO_2_ and increase the transmittance. It was also noted that the VO_2_ films remain relatively low in Δ*T*_sol_ at thicknesses close to 500 nm, and this result is most likely due to a combination of surface features and internal structure of the films.^37^ The relatively high roughness of the film surface and the formation of the oxide layer (as confirmed by the SEM images in Figure 1 and the XPS results in Figure 4) as well as the interfacial effects between the film and the substrate may affect the light transmission paths and change the optical properties of the material, causing the reflection, refraction, or scattering of the light.^38^ Also, the density of defects (such as vacancies and grain boundaries) inside the film may affect the electronic structure and the jumping behavior of VO_2_.^39^ Furthermore, for VO_2_ films, there exists a certain optical thickness that saturates the modulation of light.^40^ If this critical thickness is exceeded, then the optical interference effect may be disrupted, restricting the solar modulation capability. All these potential factors affect the overall optical response of VO_2_ films, resulting in a decrease in their solar modulation.

Compared with the common one-step annealing, we improved the crystallinity and purity of the VO_2_ films by two-step annealing, which resulted in the enhancement of their optical modulation and the reduction of the phase transition temperature. We believe that these enhancements did not originate from the prolonged total heating time involved in the two-step annealing of 3.5 h, since the effective annealing time (2 h) at higher temperature for the formation of VO_2_ (M) was unchanged. The lower temperature period (1.5 h) contributed only to the improved crystallinity and purity of VO_2_ (B). Indeed, Kang et al. have demonstrated that the longer annealing time of more than 3 h at higher temperature would lead to the degradation of the optical properties of thermochromic VO_2_ films prepared based on aqueous solution.^41^ Previous reports also concluded that the IR modulation efficiency of VO_2_ films is strongly correlated with their thickness.^42^ Considering that there is no significant difference in the thickness of the two VO_2_ film samples in the present work (refer to the SEM images of Figure 1), we suggest that the two-step annealing is the reason for the decreased transmittance and the enhanced IR/solar modulation capacity of S2. The secondary annealing further increased the crystallinity of the thin film, but also led to a higher roughness.^43^ This is also verified by the SEM images in Figure 1, in which secondary annealed S2 has a larger surface particle size. The better crystallinity led to an increased IR modulation capability, while the higher roughness slightly reduced the visible light transmittance.

## 4 Conclusions

We deposited VO_2_ films on quartz substrates by a facile sol–gel method. The changes in morphologies, structures, and infrared modulation properties of the VO_2_ films obtained by one- and two-step annealing were compared. Based on the results of our thorough characterizations, it can be concluded that VO_2_ films obtained by the two-step annealing have higher purity, better crystallinity, and enhanced infrared modulation ability, with a lower phase transition temperature, and exhibit 87% and 127% enhancement in the infrared and solar modulation capability while maintaining a high visible light transmittance. Our study demonstrated that multistep annealing is an effective means to reduce the phase transition temperature and improve the infrared modulation ability of VO_2_ film, which boosts its great potential to be applied in smart windows and related fields.

## Supporting Information Available

Supporting Information. Validation of reliability and reproducibility of results for multiple batches of samples (PDF)
