# Supplementary material for: Enhanced Optical Modulation Properties via Two-step Annealing of Sol–Gel Deposited Vanadium Dioxide Thin Films
Source: ACS Omega. 2025 Jan 20;10(4):3691–700. doi: 10.1021/acsomega.4c08910 (PMC11800157; doi:10.1021/acsomega.4c08910)

Supplementary Information

Enhanced Optical Modulation Properties via Two-step Annealing of Sol-gel Deposited Vanadium Dioxide Thin Films

Zhencheng Li[1], Jiacheng Yu[2], Yongde Xia[1], Zhuxian Yang[1], Yunbin He[2], Nannan Wang[3] and Yanqiu Zhu[1*]

[1] Department of Engineering, Faculty of Environment, Science and Economy, University of Exeter, Exeter EX4 4QF, United Kingdom

[2] School of Materials Science and Engineering, Hubei University, Wuhan 430062, China

[3] School of Resources, Environment and Materials, Guangxi University, Nanning 530004, China

The optical transmission spectra of three different batches of VO_2 thin film samples are provided. S1, S2 and S3 stand for the first (Fig. S1), second (Fig. S2) and third (Fig. S3) batches of samples, respectively. The sample serial numbers (1) and (2) in each batch refer to 1-step annealing and two-step annealing heat treatments, respectively. The results show that the optical modulation properties of the 2-step annealed samples in each batch are all enhanced compared with those of the one-step annealed samples, which proves the reproducibility and reliability of the study results. The comparison of their optical performance data is summarized in Tables S1, S2 and Fig. S4.

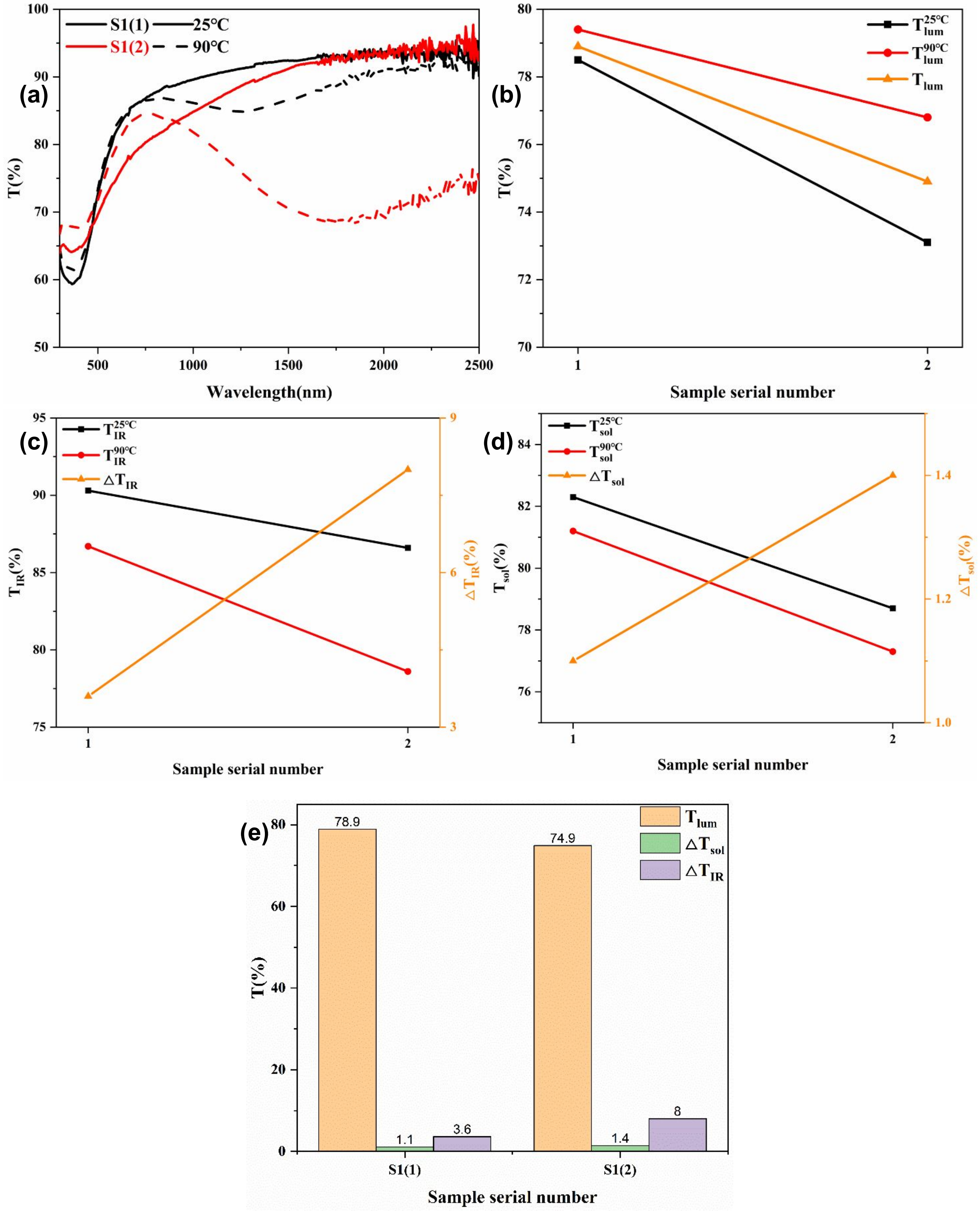

Fig. S1. (a) UV-Vis-IR optical transmittance spectra of the first batch of VO_2 film samples prepared by different annealing processes at low (25 ℃) and high (90 ℃) temperatures. (b) The integral

luminous transmittance. (c) The integral infrared-light transmittance and (d) the solar transmittance, and the corresponding modulation capacity. (e) Summary of optical data for the first batch of VO_2 film samples. The sample serial numbers 1 and 2 shown in (b), (c), (d) and (e) refer to 1-step annealing and 2-step annealing, respectively.

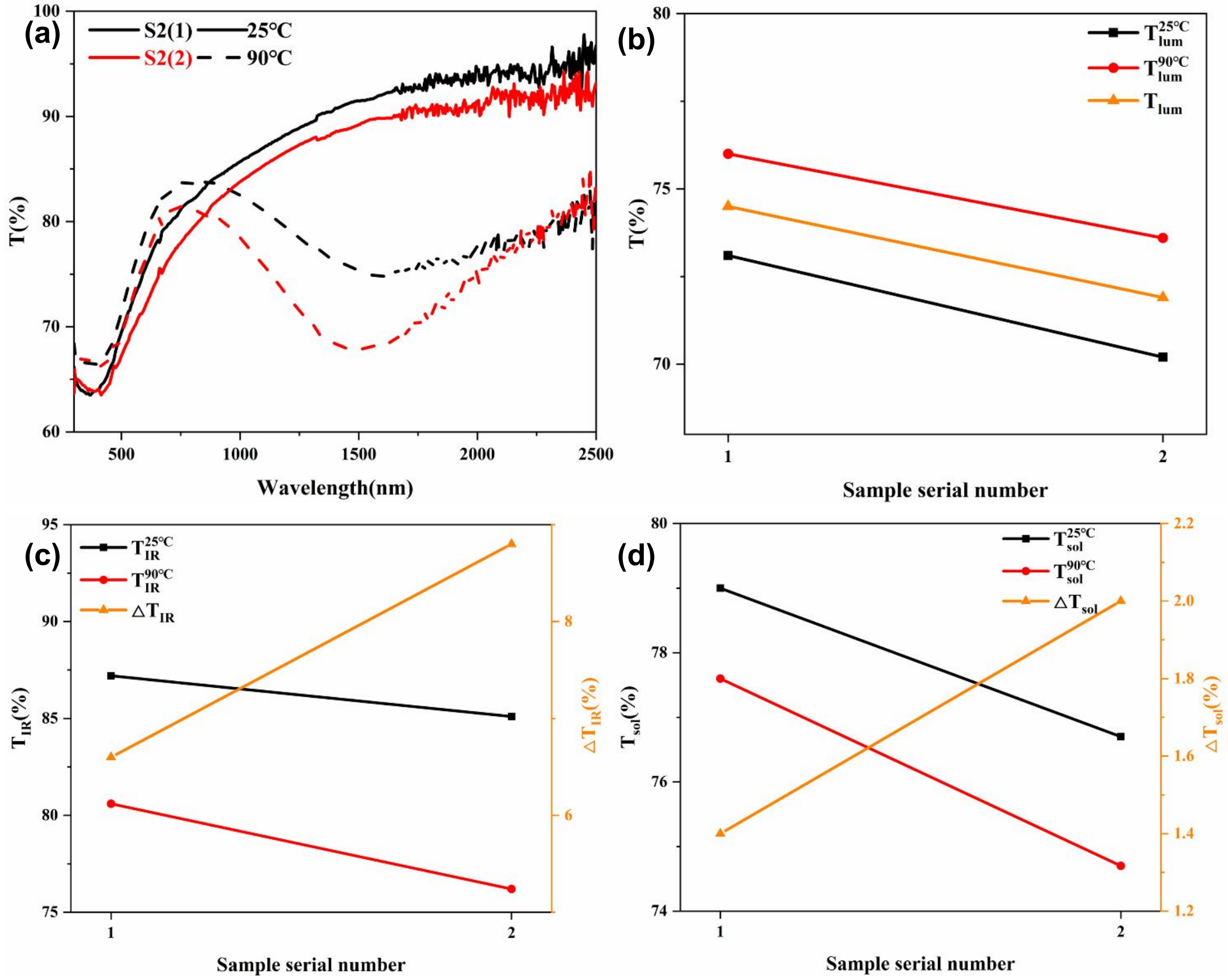

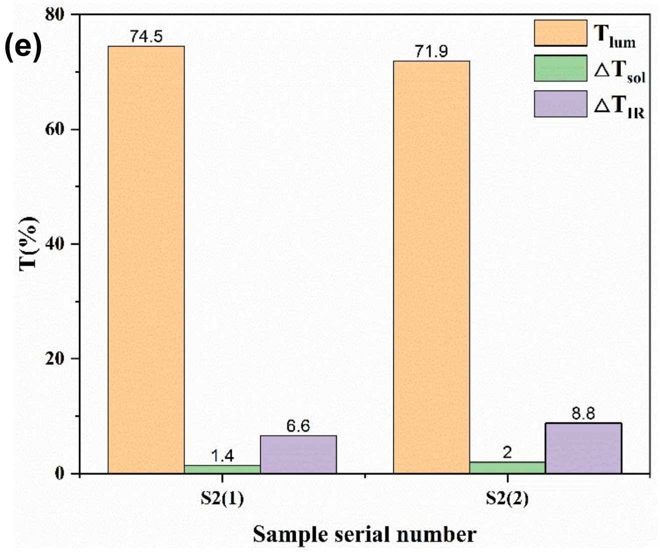

Fig. S2. (a) UV-Vis-IR optical transmittance spectra of the second batch of VO_2 film samples prepared by different annealing processes at low (25 ℃) and high (90 ℃) temperatures. (b) The integral luminous transmittance. (c) The integral infrared-light transmittance and (d) the solar transmittance, and the corresponding modulation capacity. (e) Summary of optical data for the second batch of VO_2 film samples. The sample serial numbers 1 and 2 shown in (b), (c), (d) and (e) refer to 1-step annealing and 2-step annealing, respectively.

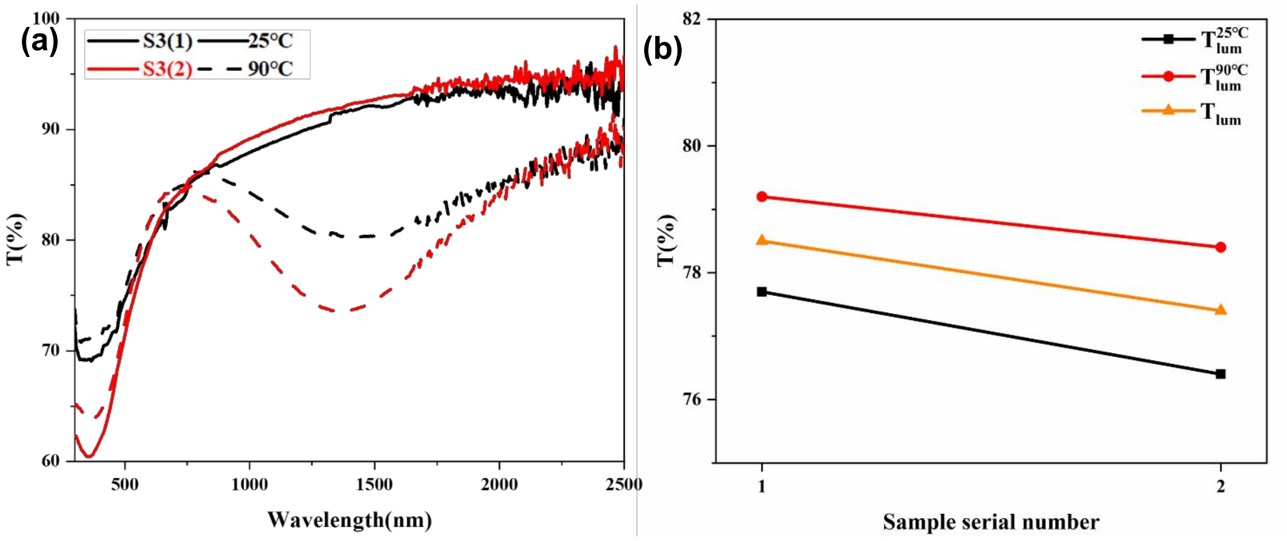

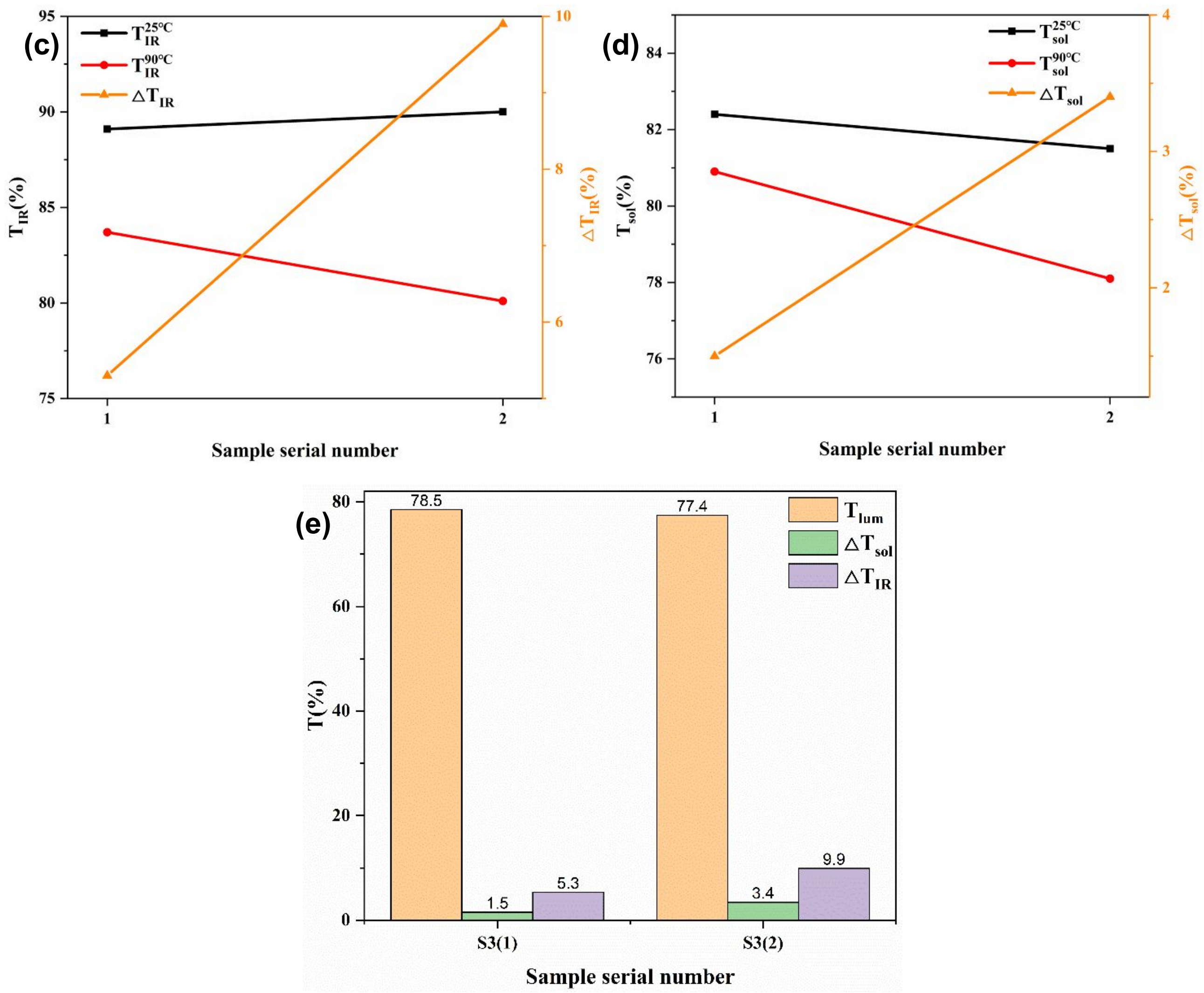

Fig. S3. (a) UV-Vis-IR optical transmittance spectra of the third batch of VO_2 film samples prepared by different annealing processes at low (25 ℃) and high (90 ℃) temperatures. (b) The integral luminous transmittance. (c) The integral infrared-light transmittance and (d) the solar transmittance, and the corresponding modulation capacity. (e) Summary of optical data for the third batch of VO_2 film samples. The sample serial numbers 1 and 2 shown in (b), (c), (d) and (e) refer to 1-step annealing and 2-step annealing, respectively.

Table S1. T_{lum}, ΔT_{sol} and ΔT_{IR} of different batches of VO_2 film samples subjected to different annealing steps.

	**S1(1)**	**S1(2)**	**S2(1)**	**S2(2)**	**S3(1)**	**S3(2)**
**T_{lum} (%)**	78.9	74.9	74.5	71.9	78.5	77.4
**ΔT_{sol} (%)**	1.1	1.4	1.4	2.0	1.5	3.4
**ΔT_{IR} (%)**	3.6	8.0	6.6	8.8	5.3	9.9

Table S2. Comparison of mean and standard deviation of T_{lum}, ΔT_{sol} and ΔT_{IR} for all one-step annealed samples and two-step annealed samples in all three batches.

	**One-step (3 samples)**	**Two-step (3 samples)**
**T_{lum} (%) _Arithmetic mean**	77.300	74.733
**T_{lum} (%) _Overall standard deviation**	1.987	2.248
**ΔT_{sol} (%) _Arithmetic mean**	1.333	2.267
**ΔT_{sol} (%) _Overall standard deviation**	0.170	0.838
**ΔT_{IR} (%) _Arithmetic mean**	5.167	8.900
**ΔT_{IR} (%) _Overall standard deviation**	1.228	0.779

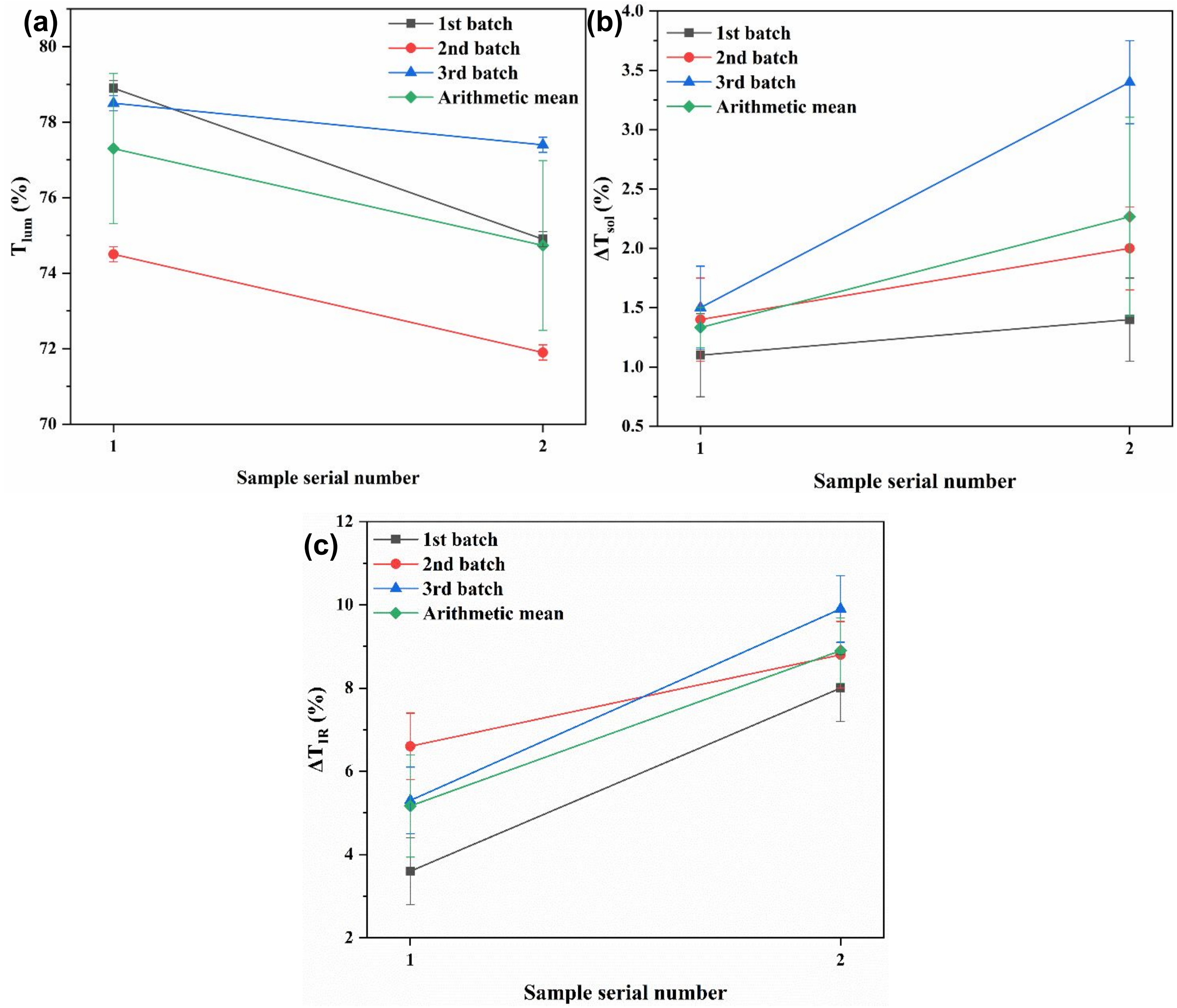

Fig. S4. Comparison of optical modulation performances of different batches of samples. (a) T_{lum}, (b) ΔT_{sol}, and (c) ΔT_{IR}.

A short video demonstrates the pouring of molten V_2O_5 into the water, which is a critical step of the sol-gel process.

(https://drive.google.com/file/d/1IFMIr71MtQuP_KRQVxLE00gasPqrHMLw/view?usp=drive_link)

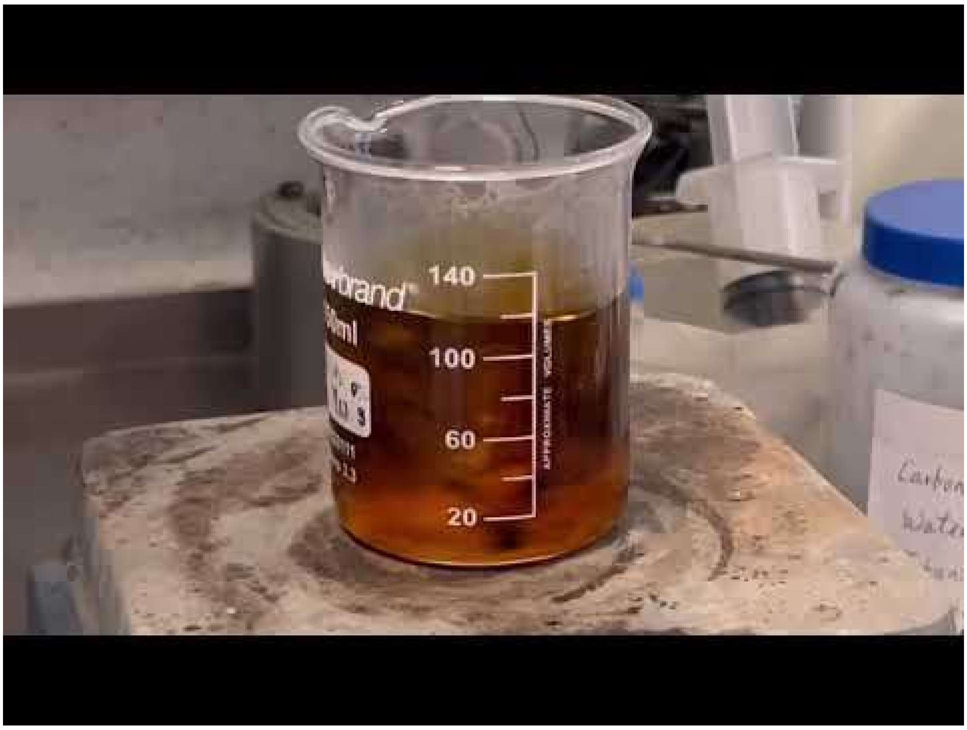

Supplement: Supplementary file 1 — ao4c08910_si_001.pdf [file ao4c08910_si_001.pdf]
